# Exploring a Single Music Therapy Session to Reduce 22q11.2 Deletion Syndrome Child and Adolescent's Outpatient Clinic‐Related Anxiety

**DOI:** 10.1111/jpc.70397

**Published:** 2026-04-27

**Authors:** Mary C. Broughton, Emma Bunzli, Susan Pagel, Lisa Burgess, Erin Davis, Karen Elliott, Vinita Prasad, Helen S. Heussler

**Affiliations:** ^1^ The University of Queensland Brisbane Queensland Australia; ^2^ Child Development Program Children's Health Queensland Hospital and Health Service Brisbane Queensland Australia; ^3^ Child, Youth & Mental Health Service Children's Health Queensland Hospital and Health Service Brisbane Queensland Australia; ^4^ Children's Health Queensland Hospital and Health Service Brisbane Queensland Australia; ^5^ Occupational Therapy and Music Therapy Department, Queensland Children's Hospital Children's Health Queensland Hospital and Health Service Brisbane Queensland Australia; ^6^ Child Development Program, Children's Health Queensland Hospital and Health Service, Centre for Children's Health Research The University of Queensland Brisbane Queensland Australia

**Keywords:** 22q11 deletion syndrome, adolescent, anxiety, caregivers, child, hospital, music therapy, outpatient clinics

## Abstract

**Aim:**

This study explored whether a single music therapy session could reduce 22q11.2 deletion syndrome (DS) children and adolescents' clinic‐related anxiety when visiting the hospital outpatient clinic for health appointments.

**Methods:**

The research employed a within‐subjects, single‐session, intervention design. The effect of the 30‐min music therapy session on children and adolescents' (henceforth children) anxiety was assessed at baseline and the end of the session. A triangulated approach examined children's anxiety through parent and child self‐report questionnaires, electrocardiograph (heart rate) and observational behavioural measures.

**Results:**

Children's self‐reported anxiety was low and did not significantly change pre‐post session, while parent/caregiver perceptions of their child's anxiety significantly decreased. Significant reduction in the frequency of anxious eye‐gaze behavior and face and bodily expressions, and an increase in non‐anxious behaviours and expressions were observed pre‐post the session. Heart rate did not significantly decrease pre‐post the music therapy session. Up to a week after the session, parents/caregivers reported a pronounced change in their child's overall anxiety after the session and a substantial improvement in their usual overall clinic experience that day.

**Conclusion:**

The findings from parent/caregiver reports and behavioural observations suggest that the music therapy session regulated the children's clinic‐related anxiety, with parents/caregivers perceiving benefits extending post‐session. However, these findings did not neatly align with children's self‐reported anxiety and heart rate data, possibly reflecting characteristics of the population and measurement challenges. Active engagement in musical experiences with a skilled facilitator, such as a music therapist, might offer a safe and potentially inexpensive non‐pharmacological approach to reducing hospital clinic‐related anxiety in 22q11.2 (DS) children and adolescents, and enhance the clinic experience.

## Brief Points

- What is already known on this topic?
  ○ High incidences of anxiety are prevalent in children and adolescents with 22q11.2 deletion syndrome (DS), a rare genetic neurodevelopmental disorder.
  ○ Frequent clinical appointments in hospital environments may exacerbate anxiety and stress for 22q11.2 DS patients and families.
  ○ Previous research with other populations suggests that engagement in music therapy may effectively ameliorate anxiety in clinical contexts.
- What this paper adds?
  ○ A single music therapy session might be an effective non‐pharmacological intervention to manage acute clinic‐related anxiety that is independent of a medical procedure in 22q11.2 DS children and adolescents.
  ○ Follow‐up parent/caregiver reports indicated a pronounced change in their child's overall anxiety following the music therapy session and a substantial improvement to their usual overall clinic experience.
  ○ This study demonstrates that child and parent/caregiver‐report, heart rate data, and observational behavioural coding capture distinct aspects related to children's anxiety and the music therapy intervention. Differences between child measures and observational and parent/caregiver perspectives highlight the complexity of assessing anxiety in this population, and underscore the importance of using multi‐method approaches to obtain a more comprehensive understanding.

## 1 Introduction

22q11.2 deletion syndrome (DS) is a rare genetic neurodevelopmental disorder caused by microdeletion of genetic material from chromosome 22 at the Q11.2 band affecting physical, mental and behavioural systems [1]. Due to the nature of symptoms, children and adolescents (henceforth children) with 22q11.2 DS often require frequent clinical appointments, potentially exacerbating the already increased incidence of anxiety prevalent in this population [2]. These children often experience significant and prolonged complex hospitalisations, including intensive care and frequent venepuncture in early infancy and childhood relating to significant congenital cardiac or gastrointestinal conditions. These children often have significant medically based trauma, and 50%–70% have anxiety. In the absence of an established approach to treating 22q11.2 DS paediatric patients' clinic‐related anxiety, we explored whether a single music therapy session could alleviate symptoms.

Previous reviews of music interventions and music therapy research suggest that active (music making) and receptive (music listening) techniques may reduce children's anxiety before, during, or after medical procedures or operations [3, 4, 5]. While few studies have been conducted in outpatient settings [6], none appear to have explored their impact on children's clinic‐related anxiety in the absence of a medical procedure. Across studies of music interventions and child anxiety associated with medical procedures, heart rate findings, children's observed behaviour and self‐reported anxiety can be mixed. This may be due to procedure‐related stress conjoined with the clinical environment. Without the additional stress of a concurrent medical procedure, a clearer decrease in children's clinic‐related anxiety, such as measured through heart rate and behavioural observations, might be expected from the music therapy. Furthermore, it is more common that child self‐reported anxiety is measured rather than parent perceptions of their child. Given that there are no validated measures for anxiety in neurodiverse children, current clinical reliability depends on parental report. To address these issues, the present study employs child and parent reports of child anxiety, behavioural observation, and heart rate measurement strategies to explore how a single music therapy session might influence children's clinic‐related anxiety.

The behavioural observation scales typically used are designed for assessing children's anxiety or distress associated with medical procedures. In contrast, few studies involve video coding of the child's behaviour in relation to the musical activities and music therapist (MT) during the intervention (see [6, 7]). This coding approach may be more suitable to examining child behaviour during music therapy because the MT purposefully seeks to access and influence the child's affective and cognitive states through the medium of music and using processes of MT‐child *musical* and *affective attunement* [7]. Of equal importance and inseparable from the musical activities is the MT, whose role is to establish rapport, trust, create a safe and supportive environment, and demonstrate responsiveness to the child's needs, enabling their active engagement and self‐expression. Theoretically, the combination of music and engagement activities within the MT‐child therapeutic dyad may work to regulate the child's emotional state [8, 9]. The MT‐child relationship is essential as it establishes an emotional context within which optimal affective states are cocreated through interpersonal interactions for emotional coregulation. Coregulation being, ‘… a bidirectional linkage of oscillating emotional channels (subjective experience, expressive behavior and autonomic physiology) between partners, which contributes to emotional and physiological stability for both partners in a close relationship.’ [8], p. 203. Thus, the music therapy approach and activities as a whole would be expected to alleviate children's anxiety, rather than any aspect within the session.

## 2 Aim

This study investigates whether a semi‐structured single 30‐min music therapy session may reduce 22q11.2 DS children's clinic‐related anxiety during outpatient visits. Using a within‐subjects intervention design, children's anxiety is assessed pre‐ and post‐session. The single music therapy session is expected to reduce children's anxiety according to child and parent‐reported anxiety, behavioural observation and heart rate measures. Specifically: children's self‐reported anxiety, parent/caregiver‐reported anxiety, and children's heart rate will decrease post‐session. Additionally, the frequency of children's anxious behaviours (e.g., eye‐gaze aversion; negative facial and bodily expressions) is expected to decrease, and non‐anxious behaviours (e.g., eye contact; positive facial and bodily expressions) will increase, post‐session.

## 3 Methods

### 3.1 Participants

Twenty‐five school‐aged children (mean age = 9.04 years, SD = 2.91 years; 11 female, 14 male) with a diagnosis of 22q11.2 DS and their parents/caregivers were recruited using opportunity sampling to participate in the research. Nine children had a psychiatric comorbidity as reported by parents/caregivers, which was anxiety in all cases. Twenty children had speech or language issues as reported by parents/caregivers: two were nonverbal, seven had delay, seven had speech issues and four did not specify. Recruitment through to data collection occurred between February and August, 2019, aligning with the children's scheduled clinic visits.

### 3.2 Ethical Considerations

The Children's Health Queensland Hospital and Health Service Human Research Ethics Committee granted approval for the research (LNR/18/QCHQ/48299); the study was conducted according to the relevant guidelines and regulations. After ethical approval, patients from the 22q11.2 DS hospital outpatient clinic in Brisbane, Queensland, Australia were invited to participate if they satisfied the eligibility criteria: child diagnosis of 22q11.2 DS, aged 6–17 years, elevated anxiety during clinic visits (reported by parent/caregiver) and no severe hearing impairment. Written informed consent was obtained from children and parents/caregivers prior to commencing the research activities.

### 3.3 Materials and Measures

#### 3.3.1 Heart Rate

Two‐lead electrocardiographs (E‐Motion Faros, MegaElectronics Pty Ltd) were used to assess participants' heart rate, as beats per minute (BPM) pre and post the intervention. Heart rate has been used as a measure of autonomic nervous system stress and anxiety response during interactive activities in children with autism spectrum disorder (ASD) [10]. For each child, mean BPM was calculated from 2 min of electrocardiograph data immediately before the music therapy session commenced (pre) and again immediately following the session (post).

#### 3.3.2 Visual Analogue Scale—Child and Parent/Caregiver Self‐Report

Child self‐reports and parent/caregiver reports regarding the child's anxiety were measured using visual analogue scales (VAS) immediately before and after the music therapy session. The VAS scale used in the present study featured a line of numerical points (0–10 cm) with corresponding facial expressions reflecting different states of anxiety (at points 0, 2, 4, 6, 8 and 10 cm). Point 0 (*not anxious*) showed a smiling face, and Point 10 (*extremely anxious*) showed a crying face, with the scale midpoint (5) being *mildly anxious*. Parents/caregivers and children marked their ratings (using an ‘x’) of the child's state of anxiety separately. Children were assisted by a research team member, which included a clinical psychologist, as needed.

#### 3.3.3 Observational Behavioural Analysis

Music therapy sessions were audio‐visually (video) recorded using a Panasonic HC‐W585M camera. Child behaviour was analysed using an observational framework comprising eye‐gaze behaviour, facial expressions, and bodily behaviours indicative of anxious or non‐anxious affective states. The framework devised for the present study incorporated components of embodied affective state, engagement, and emotion regulation, adapted from previous research with ASD children [7, 11], assessing child temperament [12, 13] and embodied emotional expression [14, 15]. Table 1 defines the target eye gaze, facial, and bodily expressions that were coded for anxious (negative) or non‐anxious (positive) behaviour categories. Behaviour frequency was measured through visual inspection of 1‐min video clips—one at the beginning and one at the end of each session, summing observations of the target behaviours in the negative and positive categories.

**TABLE 1:** Inter‐rater reliability: Intra‐class correlation (ICC) and absolute agreement results for frequencies of negative (anxious) and positive (non‐anxious) eye gaze, facial expression, and bodily behaviours in a sample of the total study participants' (*n* = 9) observational data.

Behaviour code domain	Negative (anxious)/positive (non‐anxious) affective state	Brief definition of target behaviours [references to previous research]	Inter‐rater reliability		
	(*n* = no. of observations)		ICC (95% confidence interval)	ICC rating	Absolute agreement (%)
Eye gaze	Negative (*n* = 101)	The child averts their gaze; looks toward the parent/caregiver	0.893 (0.539–0.976)	Excellent	NA
	Positive (*n* = 202)	The child holds eye contact with therapist or joins attention on a shared object [11, 13].	0.878 (0.461–0.973)	Excellent	NA
Facial expression	Negative (*n* = 43)	The child's facial expression appears anxious or fearful, such as wide and tense or closed eyes, with raised, straight or furrowed brow; lip corners may be drawn straight back or frown.	0.831 (0.23–0.962)	Excellent	NA
	Positive (*n* = 75)	The child's facial expression appears happy, indicated with smiling and a relaxed brow, or appearing concentrated or interested (on task), and eyes and brow may appear raised eyes or drawn together and squinting [7, 12, 13].	0.811 (0.244–0.956)	Excellent	NA
Bodily expression	Negative (*n* = 49)	The child shows fidgeting (with an object, gripping the chair, rocking), or body aversion to therapist or the activity, or own‐body‐directed self‐soothing behaviours (touching the face, hair, etc.), an enclosed and retreating posture, or covering eyes and ears.	NA	NA	79.39% (acceptable)
	Positive (*n* = 66)	The child shows bodily involvement in the task and free use of gesturing, such as pointing, nodding, waving, high five affirmations, body orientation to task/therapist and postural leaning in [7, 11, 12, 13, 14, 15].	0.773 (0.023–0.948)	Excellent	NA

*Note:* ICC values less than 0.40 are considered *poor*, values between 0.40 and 0.59 *fair*, values between 0.60 and 0.74 *good*, and values between 0.75 and 1.0 *excellent* indicators of inter‐rater reliability [16]. 95% confidence intervals are included in brackets following each value reported. Percentage of absolute agreement is reported where ICC values could not be calculated due to limited variance. Absolute agreement percentages above 75% are taken to indicate an acceptable level of agreement [17].

#### 3.3.4 Follow‐Up Phone Call—Music Therapy Effect on Clinic Experience

Within a week of participation, a follow‐up phone call was made to the children's parent/caregiver by a research team member. They were asked to provide ratings for two questions using a five‐point scale (1 *no change*, 2 *little change*, 3 *noticeable change*, 4 *big change*, 5 *significant change*): (1) what was the level of impact the music therapy session had on their child's overall anxiety? (2) How much did having the music therapy session help to improve their overall clinic experience that day (compared to other days)?

#### 3.3.5 Music Therapy Session Approach

The approach used Aldridge's [18] established single‐session music therapy framework, shown to relieve pre‐operative anxiety in children attending day surgery. Activities alleviate children's anxiety by creating opportunities to experience familiarity, cognitive engagement and mastery, self‐expression, as well as choice and control, through receptive and active techniques. The semi‐structured framework implemented in a one‐to‐one context allows the MT flexibility to meet the child's immediate needs and enhances responsiveness to their emotional state to alleviate anxiety.

The music therapy intervention reported here follows the outline advised by Yinger and Gooding [5]: 1. Parents provided pre‐session background information about their child's familiar songs, which were used to provide a sense of continuity between hospital and home; 2. Popular music genres were used alongside music improvisation and games; 3. The session used live music and interactive activities, which the MT adjusted according to the child's age and interests; 4. The MT‐delivered intervention utilised untuned percussion, guitar, piano and singing; 5. Intervention strategies included the same initial session structure for all participants: The greeting, usually an up‐beat song, was intended to foster the therapeutic relationship by building rapport, encouraging engagement, and gaining the child's acceptance to enter into the therapeutic process. The imitation game offered structured instrumental playing, nurturing a sense of mastery and control, using drums to help release nervous energy. Children were given many opportunities for choice, including choosing instruments and how they were played, song choice and improvising soundscapes. The soundscape improvisation created a safe space for children to express themselves non‐verbally and experience a sense of control through their choices of images, instruments, and exploring musical elements such as volume (soft/loud) and tempo (fast/slow). The farewell song was played at a moderate, calming tempo to assist in settling children to conclude the session. Throughout the session, the MT adjusted activities and her interactional style in response to the child's cues and with a goal of coregulating a non‐anxious state for the child; 6. The session occurred between clinic appointments; 7. and 8. The single‐session duration was 30 min, of which at least 90% involved music therapy; it was delivered individually to children in a clinic consultation room, designed for musical activity.

### 3.4 Procedure

After obtaining informed consent, child participants and their parents/caregivers were scheduled into a single 30‐min music therapy session, coinciding with a 22q11.2 DS clinic visit. Sessions were conducted in a designated music room at the outpatient clinic. Upon arrival, a research team member escorted participants to the room. The MT welcomed them and encouraged parents/caregivers to stay and observe the session and any changes in their child's behaviour. Parents/caregivers were also free to interact with the MT or child as necessary (or not) during the session.

Before the session, child and parent/caregiver participants completed the VAS scale individually as instructed by a research team member, who also answered any questions or assisted as needed. Another researcher applied the paediatric‐specific heart rate monitor to the child to collect pre‐ and post‐session data. A video camera started recording to capture the child's behaviour during the session, after which all members of the research team except the MT exited the room. While the session took place, the parent/caregiver completed a child background information questionnaire.

After the session, a research team member returned and stopped the video recording, then removed the heart rate measurement equipment. Child and parent/caregiver participants completed the VAS scale again individually and the questionnaires were collected. A follow‐up phone call with parents/caregivers was made up to a week after participation.

#### 3.4.1 Data Analysis Preparation

Inter‐rater reliability was assessed prior to analysing the observational behavioural data. Videos were independently coded by one researcher using the devised framework and eLAN (v. 5.2) software ([19]; see https://archive.mpi.nl/tla/elan). Data from seven child participants were excluded due to incomplete recordings or the child being out‐of‐view, leaving 18 participants for coding. Two of these coded videos were used to train a second coder/research team member by watching the videos and discussing the coding framework with Coder 1. When satisfied that they understood and could apply the coding framework, Coder 2 independently coded a random sample of nine additional recordings. For the nine recordings coded by both researchers, inter‐rater reliability was assessed using intra‐class correlation coefficients [20], and absolute agreement (percentage of agreed by total number of observations) for behaviour codes with limited variance [17]. Inter‐rater reliability for frequency data ranged from acceptable to excellent (see Table 1).

Two cases of child VAS data were missing due to participant non‐response. The HR data for eight children were not included in the analyses due to parent/caregiver‐reported current heart issues via questionnaire; four cases were missing due to technical reasons. Screening and assumption checking procedures were conducted for the different data types; the results of appropriate analyses are reported below.

## 4 Results

The results of non‐parametric exact sign tests for paired observations showed a statistically significant median decrease in parent reports of their child's anxiety pre‐post the music therapy session (median parent VAS scores: pre = 3, post = 0, *p* < 0.001). No statistically significant median change was observed for the children's self‐reported anxiety pre‐post the session (median child VAS scores: pre = 0, post = 0.2, *p* = 1).

Two‐way chi‐square test results for children's positive and negative behaviours observed pre‐post the music therapy session were statistically significant. The proportions of pre and post behaviours were different for the positive and negative behaviour types: eye gaze, *χ* ^2^ (1, *N* = 590) = 28.54, *p* = < 0.001; facial expression, *χ* ^2^ (1, *N* = 293) = 23.84, *p* = < 0.001; and bodily expression, *χ* ^2^ (1, *N* = 256) = 27.73, *p* = < 0.001. The frequencies are shown in Table 2. It can be seen that negative behaviours decreased and most positive behaviours increased pre‐post the session.

**TABLE 2:** Two‐way chi‐square test results for observational behavioural analysis frequencies.

Type of behaviour observed	Pre‐post frequency of behaviours observed		Total
	Pre	Post	
Eye gaze			
Negative			
Observed	147 (76.6%)	45 (23.4%)	192
Expected	116.8	75.2	
Positive			
Observed	212 (53.3%)	186 (46.7%)	398
Expected	242.2	155.8	
Facial expression			
Negative			
Observed	65 (73.9%)	23 (26.1%)	88
Expected	45.4	42.6	
Positive			
Observed	86 (42%)	119 (58%)	205
Expected	105.6	99.4	
Bodily expression			
Negative			
Observed	96 (76.2%)	30 (23.8%)	126
Expected	74.8	51.2	
Positive			
Observed	56 (43.1%)	74 (56.9%)	130
Expected	77.2	52.8	

Participant rankings of pre‐ and post‐session heart rate were compared using the Wilcoxon Signed‐Rank test. The median rank for pre‐session heart rate (*Mdn* = 94, range = 53) did not significantly differ from the median rank for post‐session heart rate (*Mdn* = 88, range = 40), *z* (13) = −1.09, *p* = 0.28.

When parents/caregivers were called within the week following the clinic session, they reported a *noticeable*‐to‐*big* change in their child's overall anxiety following the music therapy session (*M* = 3.77, SD = 1.36). Furthermore, parents/caregivers reported that having the music therapy session made a *big*‐to‐*significant* change or improvement in their overall clinic experience that day, compared to other days (*M* = 4.41, SD = 1.09).

## 5 Discussion

A single music therapy session might help reduce 22q11.2 DS children's anxiety, and parent/caregiver perceptions of their child's clinic‐related anxiety. Although parent/caregiver perceptions of their child's clinic‐related anxiety decreased pre‐post the session, children's anxiety self‐perceptions remained similar. The disjunct between parent/caregiver assessments of their child's anxiety significantly decreasing and the children's unchanged self‐reported anxiety is not uncommon [21]. Parents/caregivers might provide more reliable insights into their child's emotional state, as impaired emotion processing, social, and cognitive capacity is common in 22q11.2 DS children/youths [22]. The speech and language issues reported by most parents/caregivers might suggest that some children may have found the VAS scale challenging to complete, despite appearing to understand how to use it. Although anxiety is a subjective experience, parental report is the basis of current clinical reliability with no validated measures for anxiety in neurodiverse children.

Contrary to expectation, children's heart rate did not significantly decrease pre‐post the session, though the trend was in that direction and the range of scores reduced. This aligns with previous research reporting statistically significant and non‐significant results for music therapy or listening interventions before, after, and during medical procedures to reduce child distress or anxiety [3, 4, 5]. In an outpatient clinic setting [6] dissociated from a medical procedure, this study's findings suggest that a single music therapy session did not significantly reduce the heart rate physiological marker of anxiety in children. Though a reduction in anxiety‐related heart rate might have been offset by an increase due to physical engagement in the music therapy activities during the session.

The children's observed behaviour corroborates parent/caregiver perceptions, showing a decrease in anxious and an increase in non‐anxious expressions, and increased engagement in activities and with the MT. This aligns with previous music listening and therapy intervention research showing reductions in observed anxiety or distress behaviours associated with medical procedures [3, 4, 5] and in outpatient settings [6]. Findings of the relational MT‐child and musical activities video coding approach [6, 7] suggest that the children's engagement in the therapeutic relationship and activities with the MT facilitated regulation of their affective state [8, 9]. Future research will need to observe behaviour and physiology of the MT as well as the child to form any conclusions regarding coregulation. Of course, other explanations such as the potential effect of the elapse of time on the improvements observed, or engagement in preferred activities with an interested adult, are acknowledged.

Given the high rates of depression and anxiety in 22q11.2 DS children and adolescents, managing everyday and social functioning alongside health‐related difficulties is a challenge for parents/caregivers [23]. Frequent clinic visits may exacerbate underlying anxiety and manifest in disruptive behaviour, negatively influencing patient or family perceptions of the clinic experience. Since depressive and anxiety disorders increase the risk of later psychotic disorders in this population, incorporating treatment strategies into regular life is essential to promote adaptive functioning [23]. This study's findings suggest that a single music therapy session might be a viable option for managing clinic‐related anxiety for 22q11.2 DS patients. However, while parent/caregiver perceptions and child behaviour observations are positive, it is more difficult to form conclusions based on the child self‐report and heart rate measures used in this study.

## 6 Strengths and Limitations

This study suggests that a single music therapy session might help to manage clinic‐related anxiety in 22q11.2 DS child and adolescent patients. The triangulated methodology, conjoining child and parent/caregiver‐report, heart rate, and relational observational child behaviour measures, revealed how adult observer perspectives of children's anxiety may differ from measures of and from the children themselves. This highlights the importance of employing multi‐method assessments of anxiety in this population, particularly given complex characteristics that may create measurement challenges, such as relying on child self‐report. Whilst the results are promising, they are also specific to the small sample of participants and context studied, and there are opportunities for methodological advancement. For example, measuring heart rate variability (HRV), rather than heart rate, may help distinguish emotional influences from physical activity on physiological arousal. For example, low HRV is usually associated with higher levels of anxiety [24], so it may be a more precise indicator of emotion regulation. However, this may not be suitable for children with heart issues. Streamlining the behavioural video‐coding approach with checklists or automated behaviour coding tools for longer video sequences could also increase efficiency.

## 7 Conclusion

The results suggest that a single music therapy session might assist in managing anxiety for 22q11.2 DS children and adolescents in a hospital outpatient clinic environment, and independent of a medical procedure. Parents/caregivers report that these effects may extend beyond the session. However, differences between adult observer assessments of children's anxiety and those of and from the children themselves need to be reconciled with future research. Active participation in music therapy potentially offers a safe and cost‐effective non‐pharmacological approach to managing clinic‐related anxiety. Developing non‐invasive, easily implemented strategies that support young patients' psychosocial functioning within hospital settings may help mitigate the long‐term impact of medical stress, and promote more positive family engagement with health services.

## Funding

This work was funded from a generic establishment grant to support the Centre for Clinical Trials in Rare Neurodevelopmental Disorders from Queensland Health.

## Ethics Statement

This research study was conducted in full conformance with principles of the Declaration of Helsinki (DoH), Good Clinical Practice (GCP), and within the laws/regulations of Australia. The Human Research Ethics Committee, Centre for Children's Health Research, Children's Health Queensland Hospital and Health Service, provided ethical approval for the research (Approval no.: LNR/18/QCHQ/48299), which was ratified by The University of Queensland (Clearance no.: 2019000184/LNR/18/QCHQ/48299).

## Consent

Parents/caregivers gave written informed consent for de‐identified data related to them and their child from this research to be published.

## Conflicts of Interest

The authors declare no conflicts of interest.

## Data Availability

The authors have nothing to report.
